# Peptide binding predictions for HLA DR, DP and DQ molecules

**DOI:** 10.1186/1471-2105-11-568

**Published:** 2010-11-22

**Authors:** Peng Wang, John Sidney, Yohan Kim, Alessandro Sette, Ole Lund, Morten Nielsen, Bjoern Peters

**Affiliations:** 1 La Jolla Institute for Allergy and Immunology, La Jolla, USA; 2 Center for Biological Sequence Analysis, Department for Systems Biology, Technical University of Denmark, Lyngby, Denmark

## Abstract

**Background:**

MHC class II binding predictions are widely used to identify epitope candidates in infectious agents, allergens, cancer and autoantigens. The vast majority of prediction algorithms for human MHC class II to date have targeted HLA molecules encoded in the DR locus. This reflects a significant gap in knowledge as HLA DP and DQ molecules are presumably equally important, and have only been studied less because they are more difficult to handle experimentally.

**Results:**

In this study, we aimed to narrow this gap by providing a large scale dataset of over 17,000 HLA-peptide binding affinities for a set of 11 HLA DP and DQ alleles. We also expanded our dataset for HLA DR alleles resulting in a total of 40,000 MHC class II binding affinities covering 26 allelic variants. Utilizing this dataset, we generated prediction tools utilizing several machine learning algorithms and evaluated their performance.

**Conclusion:**

We found that 1) prediction methodologies developed for HLA DR molecules perform equally well for DP or DQ molecules. 2) Prediction performances were significantly increased compared to previous reports due to the larger amounts of training data available. 3) The presence of homologous peptides between training and testing datasets should be avoided to give real-world estimates of prediction performance metrics, but the relative ranking of different predictors is largely unaffected by the presence of homologous peptides, and predictors intended for end-user applications should include all training data for maximum performance. 4) The recently developed NN-align prediction method significantly outperformed all other algorithms, including a naïve consensus based on all prediction methods. A new consensus method dropping the comparably weak ARB prediction method could outperform the NN-align method, but further research into how to best combine MHC class II binding predictions is required.

## Background

HLA class II molecules are expressed by human professional antigen presenting cells (APCs) and can display peptides derived from exogenous antigens to CD4^+ ^T cells [1]. The molecules are heterodimers consisting of an alpha chain and a beta chain encoded in one of three loci: HLA DR, DP and DQ [2,3]. The DR locus can encode two beta chains DRB1 and DRB3-5 which are in linkage disequilibrium [4]. The genes encoding class II molecules are highly polymorphic, as evidenced by the IMGT/HLA database [5] which lists 1,190 known sequences of HLA class II alleles for HLA-DR, HLA-DP and HLA-DQ molecules (Table 1). Both alpha and beta chains can impact the distinct peptide binding specificity of an HLA class II molecule [6]. HLA class II peptide ligands that are recognized by T cells and trigger an immune response are referred to as immune epitopes [7]. Identifying such epitopes can help detect and modulate immune responses in infectious diseases, allergy, autoimmune diseases and cancer.

**Table 1:** Overview of human MHC class II loci, allele and polymorphism.

Locus	Gene	Chain	# of alleles
HLA-DP	HLA-DPA1	alpha	28

HLA-DP	HLA-DPB1	beta	138

HLA-DQ	HLA-DQA1	alpha	35

HLA-DQ	HLA-DQB1	beta	108

HLA-DR	HLA-DRA	alpha	3

HLA-DR	HLA-DRB1	beta	785

HLA-DR	HLA-DRB2	beta	1

HLA-DR	HLA-DRB3	beta	52

HLA-DR	HLA-DRB4	beta	14

HLA-DR	HLA-DRB5	beta	19

HLA-DR	HLA-DRB6	beta	3

HLA-DR	HLA-DRB7	beta	2

HLA-DR	HLA-DRB8	beta	1

HLA-DR	HLA-DRB9	beta	1

Information was extracted from IMGT database. HLA-DM and HLA-DO molecules are not included as they are not expressed on cell surface.

Computational predictions of peptide binding to HLA molecules are a powerful tool to identify epitope candidates. These predictions can generalize experimental findings from peptide binding assays, sequencing of naturally presented HLA ligands, and three dimensional structures of HLA peptide complexes solved by X-ray crystallography (for a review on MHC class II prediction algorithms see [8] and references herein). Several databases have been established to document the results of such experiments including Antijen [9], MHCBN [10], MHCPEP [11], FIMM [12], SYFPEITHI [13] and the Immune Epitope Database (IEDB) [14,15]. IEDB currently documents 12,577 peptides tested for binding to one of more of 158 MHC class II allelic variants of which 114 are human (HLA). It is possible to develop binding prediction methods for HLA molecules for which no experimental data are available by extrapolating what is known for related molecules [16-19]. However, the quality of these extrapolations decreases for molecules that are very different from the experimentally characterized ones, and completely *ab initio *predictions have not been successful [20]. It is therefore a major gap in knowledge that little binding data are available for HLA DP and DQ molecules, which are more difficult to work with experimentally, but are equally relevant as HLA DR molecules. Resulting from this lack of data, the vast majority of HLA class II binding predictions to date are only available for DR molecules. We here address this gap by providing a consistent, large scale dataset of binding affinities for HLA DR, DP and DQ molecules which we use to establish and evaluate peptide binding prediction tools.

It is our goal to include a variety of binding prediction algorithms in the IEDB Analysis Resource (IEDB-AR) [21], identify the best performing ones, and ideally combine multiple algorithms into a superior consensus prediction. In this study, we implemented two methods in addition to the previously incorporated ones. The first method is based on the use of combinatorial peptide libraries to characterize HLA class II molecules. Such libraries consist of mixtures of peptides of the same length, all sharing one residue at one position. Determining the affinity of a panel of such peptide libraries to an HLA molecule provides an unbiased and comprehensive assessment of its binding specificity. This approach is also time and cost effective, as the same panel of peptide libraries can be scanned for all HLA molecules of interest, and has been applied successfully for multiple applications [22-24], The second method we newly implemented was NN-align [25]. This neural network based approach combines the peptide sequence representation used in the NetMHC algorithm [26,27] that was highly successful in predicting the binding specificity of HLA class I molecules [28,29] with the representation of peptide flanking residues and peptide length used in NetMHCIIpan method [19]. Both the NN-align and the combinatorial peptide library method were evaluated in terms of their prediction performance and ability to improve a consensus prediction approach.

Finally, we wanted to address the impact of homologous peptides in our datasets on evaluating prediction results. The presence of homologous peptides in our dataset is primarily due to the strategies that were utilized in the peptide selection process. For comprehensive epitope mapping studies in individual antigens, we typically utilize 15-mer peptides overlapping by 10 residues that span entire protein sequences. Another strategy utilized to define classical binding motifs is to systematically introduce point mutations in a reference ligand to map essential residues for peptide:MHC interaction. Finally, for identified epitopes, additional variants from homologous proteins are often tested to predict potential cross-reactivity. All of these strategies introduce multiple peptides with significant sequence similarity into the dataset. This could affect the assessment of binding prediction in two distinct manners: 1) peptides in the testing set for which a homolog is present in the training may be easier to predict and thereby lead to overestimates of performance compared to real life applications; 2) the presence of multiple homologous peptides during training may bias prediction methods leading to reduced prediction performance when testing. To examine these issues, we compared evaluations with different approaches to removing similar peptides.

## Results

### Derivation and assembly of a novel MHC class II binding affinity dataset

In a previous report, we described the release of 10,017 MHC class II binding affinities experimentally measured by our group [30]. The data included measured binding affinities for a total of 17 different mouse and human allelic variants. This dataset was at the time the largest collection of homogenous MHC class II binding affinities available to the public and remains a valuable asset for the immunology research community. However, it was apparent that this dataset could be expanded and its utility improved in several regards. First, coverage of human HLA DP and DQ molecules was limited or non-existing. Secondly, for several molecules, relatively few data points existed, in spite of the fact that we and others [30,31] have shown that several hundred data points are desirable to derive accurate predictive algorithms. We have now compiled a new set of 44,541 experimentally measured, MHC class II peptide binding affinities covering 26 allelic variants (Table 2). This set includes and expands the previous set, and is the result of our general ongoing efforts to map epitopes in infectious agents and allergens. These data represent an over four fold increase in binding affinity measurements and a ~ 60% increase in allelic variant coverage. Importantly, the alleles included were selected for their high frequency in the human population (see Table 2). As a result, the combined allele frequency of this set of 26 MHC class II molecules results in >99% population coverage (Table 2). Overall, an average of 1,713 data points and 858 binders (peptides with measured IC_50 _< 1000 nM) are included for each molecule, ranging from a minimum of 577 data points for HLA DRB1*0404 and 180 binders for H-2-IAb, to the highest values of 6,427 data points and 4,519 binders for the HLA-DRB1*0101 molecule. This uniformly large number of more than 500 affinity measurements for each included allelic variant was previously found to be required to consistently generate reliable predictions [30]. To the best of our knowledge, this is the first publicly available dataset of HLA-DP and HLA-DQ binding affinities of significant size.

**Table 2:** Overview of MHC class II binding dataset utilized in the present study.

Allelic variant	# of binding affinities	**# of binders**^**1**^	% of binders	**Allele frequency**^**2**^
HLA-DPA1*0201-DPB1*0101	1399	702	0.5	16.0
HLA-DPA1*0103-DPB1*0201	1404	635	0.45	17.5
HLA-DPA1*01-DPB1*0401	1337	540	0.4	36.2
HLA-DPA1*0301-DPB1*0402	1407	621	0.44	41.6

HLA-DPA1*0201-DPB1*0501	1410	528	0.37	21.7
HLA-DQA1*0501-DQB1*0201	1658	742	0.45	11.3
HLA-DQA1*0501-DQB1*0301	1689	1023	0.61	35.1
HLA-DQA1*0301-DQB1*0302	1719	670	0.39	19.0
HLA-DQA1*0401-DQB1*0402	1701	731	0.43	12.8
HLA-DQA1*0101-DQB1*0501	1739	687	0.4	14.6
HLA-DQA1*0102-DQB1*0602	1629	974	0.6	14.6

HLA-DRB1*0101	6427	4519	0.7	5.4
HLA-DRB1*0301	1715	553	0.32	13.7
HLA-DRB1*0401	1769	978	0.55	4.6
HLA-DRB1*0404	577	396	0.69	3.6
HLA-DRB1*0405	1582	806	0.51	6.2
HLA-DRB1*0701	1745	1033	0.59	13.5
HLA-DRB1*0802	1520	591	0.39	4.9
HLA-DRB1*0901	1520	815	0.54	6.2
HLA-DRB1*1101	1794	957	0.53	11.8
HLA-DRB1*1302	1580	656	0.42	7.7
HLA-DRB1*1501	1769	909	0.51	12.2

HLA-DRB3*0101	1501	426	0.28	26.1
HLA-DRB4*0101	1521	654	0.43	41.8
HLA-DRB5*0101	1769	992	0.56	16.0
H-2-IAb	660	180	0.27	-

Total	44541	22318		
Min	577	180		
Max	6427	4519		
DP				92.6
DQ				81.6
DRB1				71.0
DRB3/4/5				70.9

Total				99.9

1. Binder defined as IC50 <1000 nM.

2. Average haplotype and phenotype frequencies for individual alleles are based on data available at dbMHC. dbMHC data considers prevalence in Europe, North Africa, North-East Asia, the South Pacific (Australia and Oceania), Hispanic North and South America, American Indian, South-East Asia, South-West Asia, and Sub-Saharan Africa populations. DP, DRB1 and DRB3/4/5 frequencies consider only the beta chain frequency, given that the DRA chain is largely monomorphic, and that differences in DPA are not hypothesized to significantly influence binding. Frequency data are not available for DRB3/4/5 alleles. However, because of linkage with DRB1 alleles, coverage for these specificities may be assumed as follows: DRB3 with DR3, DR11, DR12, DR13 and DR14; DRB4 with DR4, DR7 and DR9; DRB5 with DR15 and DR16. Specific allele frequencies at each B3/B4/B5 locus is based on published associations with various DRB1 alleles, and assumes only limited variation at the indicated locus.

### Evaluation of previously reported methods with the new dataset

In our previous evaluation of MHC class II binding prediction algorithms, we tested the performance of a large number of publicly available methods. Among those methods, ARB, SMM-align and PROPRED (based on the matrices constructed by Sturniolo et al. [16] on which also the TEPITOPE predictions are based) were the top performing ones and were incorporated into the MHC class II binding prediction component of the IEDB analysis resource [21]. Here, we re-evaluated their performance on the new dataset. As in the previous evaluation, we performed 5-fold cross validation for ARB and SMM-align and direct prediction for PROPRED over the entire data set, and quantified the performance of the various methods by calculating the AUC values using an IC_50 _cutoff of 1000 nM, as shown in Table 3 under the "current" columns. On average, the performance of the various methods was 0.784 for ARB (range 0.702 to 0.871), 0.849 for SMM-align (range 0.741 to 0.932), and 0.726 for PROPRED (range 0.600 to 0.804). Importantly, the cross-validated prediction performance for the newly included allelic variants was comparable to that of the previously included ones. Thus, the ARB and SMM-align machine learning approaches can be successfully applied to HLA DP and DQ allelic variants.

**Table 3:** Comparison of ARB, SMM-align and PROPRED's performance on current and old dataset.

Allelic variant	ARB		SMM-align		PROPRED	
	**Current^1^**	**Old^2^**	**current^1^**	**old^2^**	**current^1^**	**old^2^**

HLA-DPA1*0103-DPB1*0201	0.823		**0.921**			

HLA-DPA1*01-DPB1*0401	0.847		**0.930**			

HLA-DPA1*0201-DPB1*0101	0.824		**0.909**			

HLA-DPA1*0201-DPB1*0501	0.859		**0.923**			

HLA-DPA1*0301-DPB1*0402	0.821		**0.932**			

HLA-DQA1*0101-DQB1*0501	0.871		**0.930**			

HLA-DQA1*0102-DQB1*0602	0.777		**0.838**			

HLA-DQA1*0301-DQB1*0302	0.748		**0.807**			

HLA-DQA1*0401-DQB1*0402	0.845		**0.896**			

HLA-DQA1*0501-DQB1*0201	0.855		**0.901**			

HLA-DQA1*0501-DQB1*0301	0.844		**0.910**			

HLA-DRB1*0101	0.770	0.764	**0.798**	0.769	0.720	0.738

HLA-DRB1*0301	0.753	0.660	**0.852**	0.693	0.699	0.652

HLA-DRB1*0401	0.731	0.667	**0.781**	0.684	0.737	0.686

HLA-DRB1*0404	0.707	0.724	**0.816**	0.753	0.769	0.789

HLA-DRB1*0405	0.771	0.669	**0.822**	0.694	0.767	0.750

HLA-DRB1*0701	0.767	0.692	**0.834**	0.776	0.773	0.776

HLA-DRB1*0802	0.702	0.737	0.741	0.750	0.647	**0.768**

HLA-DRB1*0901	0.747	0.622	**0.765**	0.660		

HLA-DRB1*1101	0.800	0.731	**0.864**	0.808	0.804	0.796

HLA-DRB1*1302	0.727	0.787	**0.797**	0.695	0.600	0.584

HLA-DRB1*1501	0.763	0.700	**0.796**	0.738	0.743	0.715

HLA-DRB3*0101	0.709	0.590	**0.819**	0.677		

HLA-DRB4*0101	0.785	0.741	**0.816**	0.713		

HLA-DRB5*0101	0.760	0.703	**0.832**	0.751	0.728	0.790

H-2-IAb	0.800	0.803	**0.855**	0.746		

Average	0.784	0.706	**0.849**	0.727	0.726	0.731

Min	0.702	0.590	0.741	0.660	0.600	0.584

Max	0.871	0.803	0.932	0.808	0.804	0.796

Best prediction performance for each allelic variant was highlighted in bold.

1. The current AUC values for ARB and SMM-align were derived by cross-validation. The current AUC values for PROPRED were derived by predicting affinities for the new dataset.

2. The old AUC values were taken from previous evaluation [30].

The previously reported prediction performance data taken from [30] is also shown in Table 3 under the "old" columns. Compared to the average evaluation results reported previously, ARB (0.784 vs. 0.706) and SMM-align (0.849 vs. 0.727) showed markedly improved performance. As the training algorithms were unchanged, this most likely can be attributed to the increase in dataset sizes. In contrast, PROPRED achieved virtually the same AUC value (0.726 vs. 0.731). As the PROPRED approach is fixed and not retrained based on additional data, it is not surprising that the predictive performance on the new dataset did not differ substantially from the previously reported performance. Also, as the new data set cannot be utilized to train new PROPRED predictions, its predictions can now be generated for only a minority of the molecules considered.

### Incorporating novel prediction algorithms into the MHC class II binding prediction arsenal

In addition to the previously implemented prediction methods, we integrated two new approaches into the IEDB analysis resource. We used combinatorial peptide libraries to experimentally characterize the binding specificity of each HLA molecule for which new assays were established, including all HLA-DP and HLA-DQ allelic variants. The affinity of 180 libraries of 13-mer peptides, each sharing one amino acid residue in one of the positions from 3-11 was determined. The ability of these matrices to predict binding of individual peptides was evaluated with the entire new dataset, and the resulting AUC values are shown in Table 4 in the "ALL" column. It was found that the combinatorial library performed with AUC similar or better than the PROPRED method, which is similarly constructed based on affinity measurements for a library of single residue substitution peptides. Similar results were obtained when performance was measured with Spearman's rank correlation coefficient (Additional file 1, Table S1). This confirms that combinatorial peptide libraries are an efficient experimental approach to derive MHC class II binding profiles. Also, these predictions provide an alternative for those molecules for which the PROPRED method is not available.

**Table 4:** Cross validation prediction performances of all methods on complete and similarity reduced datasets measured with AUC.

Allelic variant	ARB		SMM-align		PROPRED		combinatorial library		NN-align		Consensus		**Consensus-best3**^**2**^	
	**ALL**	**SR^1^**	**ALL**	**SR^1^**	**ALL**	**SR^1^**	**ALL**	**SR^1^**	**ALL**	**SR^1^**	**ALL**	**SR^1^**	**ALL**	**SR^1^**

HLA-DPA1*0103-DPB1*0201	0.823	0.745	0.921	0.767			0.840	0.724	0.943	0.793	0.932	**0.809**	0.935	0.796

HLA-DPA1*01-DPB1*0401	0.847	0.746	0.930	0.767			0.833	0.704	0.947	0.802	0.938	**0.803**	0.941	0.794

HLA-DPA1*0201-DPB1*0101	0.824	0.743	0.909	0.786			0.849	0.723	0.944	0.818	0.927	0.818	0.932	**0.819**

HLA-DPA1*0201-DPB1*0501	0.859	0.709	0.923	0.728			0.867	0.729	0.956	**0.787**	0.942	0.781	0.946	0.782

HLA-DPA1*0301-DPB1*0402	0.821	0.771	0.932	0.818			0.864	0.756	0.949	0.828	0.938	**0.841**	0.941	0.830

HLA-DQA1*0101-DQB1*0501	0.871	0.741	0.930	0.783			0.809	0.728	0.945	0.805	0.933	0.809	0.942	**0.811**

HLA-DQA1*0102-DQB1*0602	0.777	0.708	0.838	0.734			0.765	0.752	0.880	0.762	0.851	0.778	0.859	**0.779**

HLA-DQA1*0301-DQB1*0302	0.748	0.637	0.807	0.663			0.698	0.616	0.851	**0.693**	0.823	0.690	0.837	0.692

HLA-DQA1*0401-DQB1*0402	0.845	0.643	0.896	0.761			0.681	0.637	0.922	0.742	0.908	0.749	0.916	**0.762**

HLA-DQA1*0501-DQB1*0201	0.855	0.700	0.901	0.736			0.586	0.620	0.932	0.777	0.917	0.774	0.923	**0.779**

HLA-DQA1*0501-DQB1*0301	0.844	0.756	0.910	0.801			0.802	0.745	0.927	0.811	0.917	0.814	0.919	**0.816**

HLA-DRB1*0101	0.770	0.710	0.798	0.756	0.720	0.692	0.739	0.697	0.843	0.763	0.810	0.759	0.820	**0.769**

HLA-DRB1*0301	0.753	0.728	0.852	0.808	0.699	0.669			0.887	0.829	0.862	0.823	0.873	**0.835**

HLA-DRB1*0401	0.731	0.668	0.781	0.721	0.737	0.711			0.813	0.734	0.799	0.735	0.804	**0.738**

HLA-DRB1*0404	0.707	0.681	0.816	0.789	0.769	0.753			0.823	0.803	0.826	0.800	0.831	**0.809**

HLA-DRB1*0405	0.771	0.716	0.822	0.767	0.767	0.742			0.870	0.794	0.847	**0.797**	0.851	**0.797**

HLA-DRB1*0701	0.767	0.736	0.834	0.796	0.773	0.750	0.762	0.729	0.869	**0.811**	0.851	0.806	0.858	0.808

HLA-DRB1*0802	0.702	0.649	0.741	0.689	0.647	0.641			0.796	0.698	0.772	0.708	0.778	**0.710**

HLA-DRB1*0901	0.747	0.654	0.765	0.696			0.572	0.553	0.810	0.713	0.801	**0.716**	0.796	**0.716**

HLA-DRB1*1101	0.800	0.777	0.864	0.829	0.804	0.779			0.900	0.847	0.880	0.850	0.885	**0.854**

HLA-DRB1*1302	0.727	0.667	0.797	0.754	0.600	0.577			0.814	0.732	0.796	0.742	0.811	**0.757**

HLA-DRB1*1501	0.763	0.696	0.796	0.741	0.743	0.703			0.852	0.756	0.820	0.756	0.827	**0.758**

HLA-DRB3*0101	0.709	0.678	0.819	0.780			0.655	0.655	0.856	0.798	0.834	0.787	0.844	**0.799**

HLA-DRB4*0101	0.785	0.747	0.816	0.762			0.697	0.691	0.870	0.789	0.844	**0.791**	0.846	0.784

HLA-DRB5*0101	0.760	0.697	0.832	0.776	0.728	0.711			0.886	0.795	0.848	0.786	0.851	**0.798**

H-2-IAb	0.800	0.775	0.855	0.830					0.858	**0.847**	0.853	0.846	0.866	**0.847**

Average	0.785	0.711	0.850	0.763	0.726	0.703	0.751	0.691	0.882	0.782	0.864	0.783	0.871	**0.786**

Min	0.702	0.637	0.741	0.663	0.600	0.577	0.572	0.553	0.796	0.693	0.772	0.690	0.778	0.692

Max	0.871	0.777	0.932	0.830	0.804	0.779	0.867	0.756	0.956	0.847	0.942	0.850	0.946	0.854

1. SR^1^stands for similarity reduced.

2. The Consensus-best3 method is based on NN-align, SMM-align and combinatorial peptide library. PROPRED was used for allelic variants when combinatorial peptide library was not available

Best prediction performance for each allelic variant was highlighted. The best performing method for "ALL" dataset was highlighted with underline while the best performing method for "SR" dataset was highlighted in bold.

The second new method we added to the IEDB analysis resource was NN-align [25]. This method differs from previous approaches in that NN-align is neural network based and can hence take into account higher order sequence correlations. Furthermore, NN-align incorporates peptide flanking residues and peptide length directly into the training of the method. This is in contrast to the SMM-align method, where the peptide flanking residues and peptide length are dealt with in an ad-hoc manner. We evaluated the performance of NN-align using the same 5-fold data separations used for the ARB and SMM-align methods. The AUC values derived from this cross validation are shown in Table 4 under the "ALL" columns and the Spearman's rank correlation coefficients were shown in Additional file 1, Table S1. The NN-align method stands out as having by far the best performance, with an average AUC value of 0.882 and average Spearman's rank correlation coefficient of 0.758.

### A novel homology reduction approach for unbiased cross validation

Some peptides in our dataset have significant homology to each other which could bias the cross-validation results if similar peptides are present in both the training and the testing sets. Previous studies have attempted to address this issue and several strategies have been proposed to generate sequence similarity reduced datasets for cross-validation purpose. One such approach is to remove similar peptides from the entire dataset [32]. We call this a 'random selection' strategy as the order in which peptides are removed is not defined. We applied the algorithm to our dataset and for any two peptides that shared an identical 9-mer core region, or that had more than 80% overall sequence identity, one peptide was removed. The results are shown in Additional file 1, Table S2 and highlight that this strategy selected a different number of peptides in repeated runs. To avoid this, we applied a Hobohm 1 like selection strategy that deterministically selects a set of peptides, and also maximizes the number of peptides included in the data. This was done by a forward selection procedure described in the methods section. Briefly, for each peptide the number of similar peptides was recorded and peptides were sorted according to this number. Peptides were selected from this ordered list starting with those with the smallest number of similar peptides. If a peptide was encountered for which a similar matching one was already selected, it was discarded. As shown in Additional file 1, Table S2, this strategy indeed resulted in a stable selection of peptides and always selected a higher number of peptides than the random selection algorithm.

Using the forward selection algorithm, we derived sequence Similarity Reduced (SR) datasets and used them in five-fold cross validation to evaluate the performance of our panel of MHC class II binding prediction tools. The results are shown in Table 4 under columns titled SR. Clearly, reducing sequence similarity had a significant impact on the observed classifier performance, which is consistent with previous findings [32]. At the same time, the order of performance of the different prediction methods was unchanged when using the reduced dataset, with NN-align performing the best, SMM-align second, ARB third, and PROPRED and the combinatorial libraries last. The order of performance determined by Spearman's rank correlation coefficient analysis (Additional file 1, Table S1) was largely identical except that ARB and PROPRED switched position. The largest drop in performance was observed for NN-align and SMM-align, where the average AUC value was reduced by 0.100 and 0.087 (0.151 and 0.130 in terms of Spearman's rank correlation coefficient) when tested with similarity reduced datasets, respectively. The smallest reduction was observed for PROPRED with an average AUC reduction of 0.023 (0.036 in terms of Spearman's rank correlation coefficient) followed by the combinatorial peptide library with a reduction in AUC of 0.060 (0.099 in terms of Spearman's rank correlation coefficient). As the latter two methods do not utilize the training dataset to make their prediction, it is expected that they show less of a drop in performance than the others. The fact that a reduction in performance was observed at all indicates that removing similar peptides from the testing set alone makes the prediction benchmark harder. This can be explained by the fact that homologous peptides removed because they are single residue substitutions of known epitopes or reference ligands are often 'easy' to predict, as they carry strong and straightforward signals to discover binding motifs.

### Training with peptides of significant sequence similarity doesn't negatively influence the prediction of unrelated sequences

An important question arising from the sequence similarity reduction and cross validation evaluation is whether inclusion of similar sequences will have a negative impact on the prediction of unrelated sequences. An excessive amount of peptides with similar sequences may bias a classifier such that the performances on sequences without significant similarity to the training data are negatively influenced. This was demonstrated in [32] in which a classifier displayed better performance than others when evaluated on a dataset that contained similar sequences, but which completely failed when evaluated on a dataset with no homology between peptides. It is unclear though how relevant this finding is in practice, specifically as the inclusion of single residue substitutions can contain particularly useful information demonstrated by the fact that this is how the MHC binding motifs were originally defined [33].

**Figure 1:**
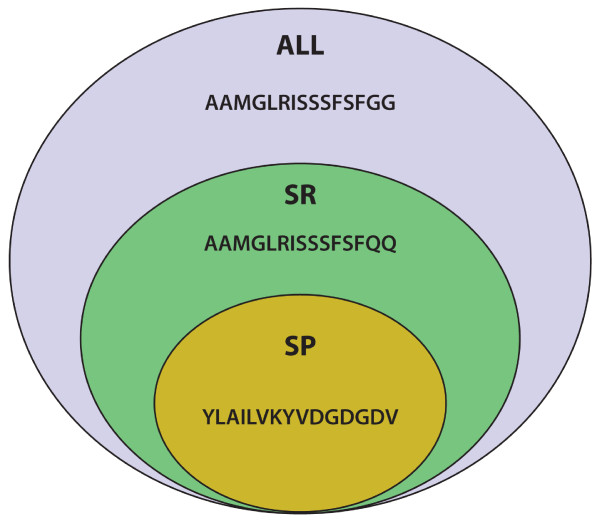
**A Venn diagram illustrating the relationship among "ALL", "SR' and "SP" datasets**. The simulated dataset illustrated the superset relationships among the "ALL", "SR" and "SP" sets. The "ALL" dataset contains all three peptides. The "SR" dataset contains two peptides with one of the similar peptide being removed and the "SP" dataset only contains a single peptide that shares no similarity with any other peptides.

We developed a simple strategy to test if the inclusion of homologous peptides in the training data can affect the prediction of unrelated peptides. For each allelic variant, we selected a subset of singular peptides (SP) set, which share no sequence similarity with any other peptides in the set (Figure 1). The similarity reduced (SR) set is a superset of the SP set, which in addition to the SP peptides also contains one peptide from each cluster of similar peptides. For each peptide in the SP set, there exist two blinded binding predictions obtained in the previous cross validations: One where the training set included all peptides including homologs (the ALL set), the other where only non-homologous peptides were included in the training (the SR set). By comparing the performance of the two predictions, we evaluated if inclusion of homologous peptides in the training negatively impacts the prediction of non-homologous peptides. We performed this test on all implemented machine learning methods with similar results, and are showing the resulting AUCs for the top performing method NN-align in Table 5. On average, the performance of methods trained including homologues was higher than methods trained leaving out those peptides. While the difference is not significant (paired two tailed t-test, p-value = 0.259), this alleviates concerns for the tested methods that predictions will actually get worse when including homologous peptides in the training. Thus, it is advisable that the ultimate classifiers for public use should be trained using all available binding data.

**Table 5:** Prediction performance on singular peptide set (SP) using training sets with and without homologs.

Allelic variant	SR AUC	ALL AUC	AUC reduction^1^	# peptide reduction^2^	% peptide reduction^3^
HLA-DPA1*0103-DPB1*0201	0.787	0.797	0.010	801	0.571

HLA-DPA1*01-DPB1*0401	0.809	0.801	-0.008	797	0.596

HLA-DPA1*0201-DPB1*0101	0.764	0.735	-0.029	795	0.568

HLA-DPA1*0201-DPB1*0501	0.587	0.640	0.053	824	0.584

HLA-DPA1*0301-DPB1*0402	0.744	0.772	0.028	805	0.572

HLA-DQA1*0101-DQB1*0501	0.850	0.821	-0.029	1155	0.664

HLA-DQA1*0102-DQB1*0602	0.667	0.719	0.052	1036	0.636

HLA-DQA1*0301-DQB1*0302	0.569	0.756	0.187	1123	0.653

HLA-DQA1*0401-DQB1*0402	0.632	0.551	-0.081	1116	0.656

HLA-DQA1*0501-DQB1*0201	0.587	0.652	0.065	1069	0.645

HLA-DQA1*0501-DQB1*0301	0.764	0.766	0.002	1087	0.644

HLA-DRB1*0101	0.777	0.781	0.004	2923	0.455

HLA-DRB1*0301	0.782	0.786	0.004	579	0.338

HLA-DRB1*0401	0.682	0.709	0.027	548	0.310

HLA-DRB1*0404	0.805	0.818	0.013	103	0.179

HLA-DRB1*0405	0.765	0.748	-0.017	533	0.337

HLA-DRB1*0701	0.793	0.810	0.017	570	0.327

HLA-DRB1*0802	0.672	0.622	-0.050	503	0.331

HLA-DRB1*0901	0.669	0.651	-0.018	478	0.314

HLA-DRB1*1101	0.809	0.799	-0.010	590	0.329

HLA-DRB1*1302	0.712	0.733	0.021	510	0.323

HLA-DRB1*1501	0.712	0.719	0.007	598	0.338

HLA-DRB3*0101	0.829	0.838	0.009	514	0.342

HLA-DRB4*0101	0.762	0.745	-0.017	510	0.335

HLA-DRB5*0101	0.774	0.798	0.024	571	0.323

H-2-IAb	0.816	0.833	0.017	114	0.173

Average	0.737	0.748	0.011	779	0.444

The "ALL" column indicates 5-fold cross validation performance of this subset trained with entire dataset. The "SR" indicates 5-fold cross validation performance of this subset trained with sequence similarity reduced dataset.

1. AUC reduction = AUC all - AUC SR

2. # peptide reduction = # peptide all - # peptide SR

3. % peptide reduction = (# peptide all - # peptide SR)/# peptide all

### A consensus approach of selected methods outperforms a generalized consensus approach and individual methods

In our previous study, a median rank based consensus approach gave the best prediction performance. In this study, we updated the consensus approach with the new methods (NN-align and combinatorial peptide library) and evaluated its performance on the similarity reduced as well as entire dataset (Table 4). The result showed that while the consensus method remains a competitive approach, it does not outperform the best available individual approach NN-align (paired one tailed t-test, p-value = 0.135) on the similarity reduced dataset.

We next investigated optimized approaches for deriving consensus predictions. We reasoned that simply increasing the number of methods included in a consensus prediction might not be optimal, especially if certain methods are underperforming, or simply if multiple methods are conceptually redundant (based on identical or similar approaches). To determine the benefit of including individual methods in the consensus, we tested the performance of the consensus approach while removing each of the five methods (Additional file 1, Table S3) using the similarity-reduced dataset. The results indicated that removing NN-align, SMM-align, the combinatorial peptide library and PROPRED reduced prediction performance. In contrast, removing ARB actually had a positive impact on consensus performance. Based on this, we tested the performance of a consensus approach on the SR dataset utilizing NN-align, SMM-align and the combinatorial library, or substituted PROPRED for the combinatorial library for those alleles for which it is not available (labeled consensus-best3). The resulting average AUC on the SR set (0.786) is significantly improved over consensus using all methods (paired, one sided t-test, p-value = 0.033). Also, the prediction performance of consensus-best3 in comparison to NN-align is significantly better in the SR set (paired, one sided t-test, p-value = 0.0034). When performance was measured with Spearman's rank correlation coefficient, very similar results were obtained though the performance of NN-align and consensus-best3 were virtually identical on the SR set. Thus, a combination of selected subsets of methods for a consensus could achieve better performance than the naïve consensus approach in which all methods were utilized.

### Inclusion of the novel dataset into the IEDB and integration of the algorithms in the IEDB analysis resource

We have updated the MHC class II portion of the IEDB analysis resource http://tools.immuneepitope.org/analyze/html/mhc_II_binding.html to reflect the progress in data accumulation and algorithm development. There are now six algorithms available to predict MHC class II epitope: the previously established ARB, SMM-align and PROPRED methods, the newly established combinatorial library and NN-align predictions, and the combined consensus approach. The ARB algorithm has been re-implemented in Python to allow better integration with the website and future development. The machine learning based approaches (ARB, NN-align and SMM-align) have been retrained with the complete dataset described in this article to provide improved performance. The collection of algorithms has also been implemented as a standalone command line application that provides identical functionality as the website. This package can be downloaded from the IEDB analysis resource along with the MHC class II binding affinity datasets, the prediction scores, and the combinatorial peptide library matrices.

## Discussion and Conclusions

Computational algorithms to predict epitope candidates have become an essential tool for genomic screens of pathogens for T cell response targets [34-37]. The majority of these algorithms rely on experimental binding affinities to generate predictive models. The data presented in this study provides a large scale and homogenous dataset of experimental binding affinities for HLA class II molecules, along with a comprehensive evaluation of prediction performances for a number of algorithms. The binding dataset made available here is about four-fold larger than the one in our previous report [30]. The increased number of peptides per allele resulted in a significantly improved performance of machine learning methods, ARB and SMM-align. This reinforces the idea that the prediction performance of a machine learning method is greatly dependent on the amount of learning data available.

This present dataset is not only significantly larger than what was previously available, but also for the first time covers HLA-DP and HLA-DQ molecules in depth. Lack of data for these alleles was identified in previous studies as one of the challenges facing HLA class II binding predictions [30,31]. The significant increase (i.e. over 40%) in the number of allelic variants results in a > 99% population coverage which could be very valuable for the development of T-cell epitope based vaccine. This dataset will also be useful in improving pan-like approaches that take advantage of binding pocket similarities among different MHC molecules to generate binding predictors for allelic variants without binding data [19].

We added two new methods to our panel of prediction algorithms. Combinatorial peptide libraries were used to experimentally characterize HLA class II alleles for which no PROPRED predictions were available. Data from such libraries have successfully been used to predict proteasomal cleavage [22], TAP transport [23] and MHC class I binding [24]. The performance of the libraries for class II predictions was comparable to that of PROPRED, and in general inferior to the machine learning approaches. The main value of the combinatorial library approach lies in its experimental efficiency, and in that its predictions can be considered completely independent of those from machine learning algorithms. The combinatorial library approach increases its value when combined with machine learning methods for consensus prediction approaches.

The second method added was NN-align, which showed a remarkably high prediction performance in the benchmark. This repeats the dominating performance of the related NetMHC prediction methods in a number of recent MHC class I prediction benchmarks [28,29,38].

One of the challenges for evaluating the MHC class II binding prediction performances is how to deal with the presence of homologous peptides in the available data [32]. One concern is that peptides in the testing set for which a homolog is present in the training data may lead to artificially high prediction performances. To address this, we generated sequence similarity reduced dataset from the entire available data using a forward selection approach such that no homologous peptides are present in the subset. The prediction performance on this similarity reduced dataset shows that the absolute AUC values of the compared methods is indeed significantly lower than that of the entire dataset. However, the rank-order of the different prediction methods was largely unchanged between datasets. This leads us to conclude that 1) the impact of homologous peptides shared between training and testing datasets has a minor impact on rankings of prediction methods at least for large scale datasets, but should nevertheless be corrected for. 2) Prediction performance comparisons between different methods cannot be made based on absolute AUC values unless both training and testing datasets are identical.

A second concern when dealing with homologous peptides in the training dataset is that the presence of a large number of similar peptides may bias the classifier such that the prediction performance of unrelated peptides is negatively affected. We performed a direct comparison of the predictive performance on novel peptides based on classifiers trained in the presence and absence of similar peptides. The comparison showed that there is a performance gain for classifiers trained with the larger dataset including similar peptides. Thus we recommend that classifiers created for end user applications should be trained with all available data to gain maximum predictive power for epitope identification.

Constructing meta-classifiers is a popular approach to improve predictive performance. We previously reported a median rank based consensus approach that outperforms individual MHC class II binding prediction methods. With the addition of new methods, we found that consensus methods including all available methods failed to outperform the best available individual method. On the other hand, when only methods that contributed positively to the consensus approach were included, the consensus approach outperformed the best individual method (0.786 vs. 0.782) on the "SR" dataset. The absolute values of improved average AUC is much smaller than that was reported in our previous study (0.004 vs. 0.033). This suggested that simple median rank based approach is less effective as individual method's performance improves and more sophisticated consensus approaches are needed to capitalize on a large array of MHC class II binding prediction methods. Also, the best individual method (NN-align) still outperformed the consensus with selected methods when they were tested with the "ALL" dataset. Since there are significant peptide similarities in the "ALL" dataset, this could be due to overfitting. We plan to systematically examine how to best construct consensus predictions for MHC binding in the future, building on work done by us and others in the past [30,39,40].

## Methods

### Positional scanning combinatorial libraries and peptide binding assays

The combinatorial libraries were synthesized as previously described [24,41]. Peptides in each library are 13-mers with Alanine residues in positions 1, 2, 12 and 13. The central 9 residues in the peptides are equal mixtures of all 20 naturally occurring residues except for a single position per library which contains a fixed amino acid residue. A total of 180 libraries were used to cover all possible fixed residues at all positions in the 9-mer core. The IC50 values for an example peptide library (HLA-DPA1*0103-DPB1*0201) are shown in Additional file 1, Table S4.

The binding assay methods for MHC class II molecules in general [42,43] as well as HLA-DP [44] and HLA-DQ [45] molecules have been described in detail previously.

### Deriving scoring matrix for positional scanning combinatorial peptide libraries

IC50 values for each mixture were standardized as a ratio to the geometric mean IC50 value of the entire set of 180 mixtures, and then normalized at each position so that the value associated with the optimal value at each position corresponds to 1. For each position, an average (geometric) relative binding affinity (ARB) was calculated, and then the ratio of the ARB for the entire library to the ARB for each position was derived. The final results are a set of 9 × 20 scoring matrices were used to predict the binding of novel peptides to MHC molecules by multiplying the matrix values corresponding to the sequence of 9-mer cores in the peptide of interest. An example scoring matrix (HLA-DPA1*0103-DPB1*0201) is shown in Additional file 1, Table S5.

### Generation of similarity reduced datasets for cross validation

Several previous studies have proposed measurements to determine peptide similarity [32,46-49]. Here we adopted the similarity measure described by El-Manzalawy et al. [32]. Two peptides were defined as similar if they satisfied one of the following conditions: (1) The two peptides share a 9-mer subsequence. (2) The two peptides have more than 80% sequence identity. The sequence identity was calculated as follows. For peptide p1 with length L_1 _and peptide p2 with length L_2_, all non-gap alignments between p1 and p2 were examined. The number of identical residues in each alignment was compared and the maximum M was taken as the number of identical residues between the two peptides. The sequence identity was then calculated as M/min(L_1_, L_2_).

In order to derive the similarity reduced (SR) dataset, we first partitioned the dataset into binder and non-binder using an IC_50 _cutoff of 1000 nM. The cutoff of 1000 nM was chosen for its biological relevance as a previous study showed that a cutoff of 1000 nM captured near 97% DR-restricted epitopes [50]. For each peptide in a partition, we first determined its similarity with the rest of peptides in the dataset and the number of peptides sharing similarity with each peptide (N_similarity_) was recorded. We then sorted the peptides according to their N_similarity _in ascending order and stored the sorted peptides in a list L_all_. The forward step-wise Hobohm 1 algorithm [51] consisting of the following three steps was next applied to generate a similarity reduced:

1. Start with an empty dataset, Set_SR,_.

2. The peptide on top of L_all _(P_top_) is removed from L_all _and compared with all peptides in Set_SR_. If the peptide P_top _is not similar with any peptide in Set_SR_, then P_top _is stored in Set_SR _otherwise P_top _is discarded.

3. Repeat step 2 until L_all _is empty.

The peptides selected by this procedure for the binder and non-binder partitions were then combined to generate the final SR dataset.

In order to test whether the inclusion of homologous peptides in the training data can affect the prediction of unrelated peptides, we generated a singular peptides (SP) set. For each allelic variant, we selected a subset of peptides, which share no sequence similarity with any other peptides in the set.

The three sets of peptides used in the study have a simple superset relationship in that the "ALL" set is a superset of "SR" set and the "SR" set is a superset of the "SP" set. The relationship was further illustrated in Figure 1.

### Cross validation and performance evaluation with ROC

Two types of performance evaluation were carried out. For the combinatorial library and the PROPRED predictions which are not trained on peptide binding data, the entire dataset was used to measure prediction performance. For the ARB, SMM-align and NN-align predictions which require peptide binding data for training, five-fold cross validations were performed to measure classifier performance. For the consensus approach, the predictions were generated for each method as described above and then combined to generate the consensus.

Receiver operating characteristic (ROC) curves [52] were used to measure the performance of MHC class II binding prediction tools. For binding assays, the peptides were classified into binders (experimental IC_50 _< 1000 nM) and nonbinders (experimental IC_50_≥1000 nM) as described previously [30]. For a given prediction method and a given cutoff for the predicted scores, the rate of true positive and false positive predictions can be calculated. An ROC curve is generated by varying the cutoff from the highest to the lowest predicted scores, and plotting the true positive rate against the false positive rate at each cutoff. The area under ROC curve is a measure of prediction algorithm performance where 0.5 is random prediction and 1.0 is perfect prediction. The plotting of ROC curve and calculation of AUC were carried out with the ROCR [53] package for R [54]. In addition, the predictive performance was also evaluated via Spearman's rank correlation coefficient.

## Authors' contributions

PW, JS, YK, AS, OL, MN and BP conceived and designed the experiments. PW and JS performed the experiments. PW, JS, MN and BP analyzed the data. PW, JS, YK, AS, OL, MN and BP wrote the paper. All authors read and approved the final manuscript.

## Supplementary Material

Additional file 1 **Supplementary Tables**. Description: five supplementary tables that contain additional analysis described in the paper. Click here for file
