# Supplementary material for: Peptide binding predictions for HLA DR, DP and DQ molecules
Source: BMC Bioinformatics. 2010 Nov 22;11:568. doi: 10.1186/1471-2105-11-568 (PMC2998531; doi:10.1186/1471-2105-11-568)
Supplement: Additional file 1 — Supplementary Tables. Description: five supplementary tables that contain additional analysis described in the paper. [file 1471-2105-11-568-S1.DOC]

**Table S1.** **Cross validation prediction performances of all methods on complete and similarity reduced datasets measured with Spearman’s rank correlation coefficient.**

Best prediction performance for each allelic variant was highlighted. The best performing method for "ALL" dataset was highlighted with underline while the best performing method for "SR" dataset was highlighted in bold.

| Allelic variant | ARB | | SMM-align | | PROPRED | | Combinatorial library | | NN-align | | Consensus | | Consensus-best3^2^ | |
| --- | --- | --- | --- | --- | --- | --- | --- | --- | --- | --- | --- | --- | --- | --- |
|  | ALL | SR^1^ | ALL | SR^1^ | ALL | SR^1^ | ALL | SR^1^ | ALL | SR^1^ | ALL | SR^1^ | ALL | SR^1^ |
| HLA-DPA1*0103-DPB1*0201 | 0.636 | 0.504 | 0.831 | 0.577 |  |  | 0.665 | 0.438 | 0.872 | 0.637 | 0.843 | **0.639** | 0.855 | 0.626 |
| HLA-DPA1*01-DPB1*0401 | 0.634 | 0.517 | 0.829 | 0.588 |  |  | 0.650 | 0.421 | 0.871 | 0.642 | 0.850 | **0.648** | 0.862 | 0.642 |
| HLA-DPA1*0201-DPB1*0101 | 0.683 | 0.551 | 0.841 | 0.641 |  |  | 0.720 | 0.473 | 0.898 | **0.701** | 0.866 | **0.701** | 0.877 | 0.697 |
| HLA-DPA1*0201-DPB1*0501 | 0.634 | 0.444 | 0.773 | 0.522 |  |  | 0.676 | 0.448 | 0.854 | 0.612 | 0.818 | 0.607 | 0.830 | **0.614** |
| HLA-DPA1*0301-DPB1*0402 | 0.645 | 0.588 | 0.855 | 0.678 |  |  | 0.734 | 0.553 | 0.889 | 0.724 | 0.871 | **0.738** | 0.875 | 0.715 |
| HLA-DQA1*0101-DQB1*0501 | 0.687 | 0.481 | 0.809 | 0.562 |  |  | 0.586 | 0.430 | 0.858 | **0.640** | 0.825 | 0.626 | 0.845 | 0.626 |
| HLA-DQA1*0102-DQB1*0602 | 0.574 | 0.440 | 0.680 | 0.505 |  |  | 0.507 | 0.508 | 0.743 | 0.571 | 0.701 | 0.591 | 0.711 | **0.597** |
| HLA-DQA1*0301-DQB1*0302 | 0.558 | 0.394 | 0.676 | 0.501 |  |  | 0.468 | 0.361 | 0.775 | **0.561** | 0.728 | 0.550 | 0.751 | **0.561** |
| HLA-DQA1*0401-DQB1*0402 | 0.661 | 0.360 | 0.778 | 0.582 |  |  | 0.415 | 0.340 | 0.832 | 0.578 | 0.802 | 0.578 | 0.816 | **0.590** |
| HLA-DQA1*0501-DQB1*0201 | 0.660 | 0.424 | 0.787 | 0.527 |  |  | 0.260 | 0.307 | 0.853 | **0.603** | 0.816 | 0.589 | 0.834 | 0.600 |
| HLA-DQA1*0501-DQB1*0301 | 0.686 | 0.570 | 0.814 | 0.667 |  |  | 0.596 | 0.534 | 0.847 | **0.692** | 0.829 | 0.682 | 0.835 | 0.690 |
| HLA-DRB1*0101 | 0.560 | 0.471 | 0.620 | 0.565 | 0.480 | 0.446 | 0.477 | 0.429 | 0.706 | 0.589 | 0.647 | 0.582 | 0.663 | **0.597** |
| HLA-DRB1*0301 | 0.532 | 0.475 | 0.680 | 0.624 | 0.398 | 0.364 |  |  | 0.763 | **0.671** | 0.715 | 0.659 | 0.725 | 0.662 |
| HLA-DRB1*0401 | 0.471 | 0.358 | 0.552 | 0.457 | 0.466 | 0.424 |  |  | 0.624 | 0.479 | 0.591 | 0.487 | 0.595 | **0.493** |
| HLA-DRB1*0404 | 0.405 | 0.352 | 0.604 | 0.554 | 0.513 | 0.497 |  |  | 0.595 | 0.567 | 0.623 | 0.576 | 0.627 | **0.578** |
| HLA-DRB1*0405 | 0.581 | 0.481 | 0.672 | 0.594 | 0.561 | 0.518 |  |  | 0.757 | 0.642 | 0.720 | **0.649** | 0.729 | 0.645 |
| HLA-DRB1*0701 | 0.568 | 0.503 | 0.704 | 0.648 | 0.589 | 0.548 | 0.521 | 0.480 | 0.770 | **0.685** | 0.738 | 0.673 | 0.744 | 0.677 |
| HLA-DRB1*0802 | 0.385 | 0.289 | 0.458 | 0.385 | 0.291 | 0.281 |  |  | 0.578 | **0.427** | 0.523 | 0.422 | 0.535 | **0.427** |
| HLA-DRB1*0901 | 0.484 | 0.312 | 0.544 | 0.410 |  |  | 0.144 | 0.106 | 0.618 | **0.453** | 0.601 | 0.443 | 0.596 | 0.447 |
| HLA-DRB1*1101 | 0.613 | 0.571 | 0.720 | 0.662 | 0.589 | 0.549 |  |  | 0.793 | **0.710** | 0.753 | 0.705 | 0.753 | 0.707 |
| HLA-DRB1*1302 | 0.471 | 0.364 | 0.586 | 0.523 | 0.220 | 0.189 |  |  | 0.636 | 0.512 | 0.595 | 0.516 | 0.614 | **0.530** |
| HLA-DRB1*1501 | 0.553 | 0.443 | 0.617 | 0.522 | 0.496 | 0.419 |  |  | 0.708 | **0.557** | 0.653 | 0.552 | 0.660 | 0.555 |
| HLA-DRB3*0101 | 0.443 | 0.395 | 0.602 | 0.552 |  |  | 0.307 | 0.320 | 0.682 | 0.604 | 0.648 | 0.591 | 0.658 | **0.605** |
| HLA-DRB4*0101 | 0.580 | 0.529 | 0.646 | 0.565 |  |  | 0.405 | 0.394 | 0.758 | **0.633** | 0.699 | 0.624 | 0.709 | 0.617 |
| HLA-DRB5*0101 | 0.548 | 0.442 | 0.687 | 0.597 | 0.487 | 0.462 |  |  | 0.781 | **0.634** | 0.722 | 0.624 | 0.725 | 0.631 |
| H-2-IAb | 0.549 | 0.486 | 0.660 | 0.628 |  |  |  |  | 0.659 | **0.664** | 0.658 | 0.653 | 0.678 | **0.664** |
| Average | 0.569 | 0.452 | 0.693 | 0.563 | 0.463 | 0.427 | 0.508 | 0.409 | 0.758 | **0.607** | 0.724 | 0.604 | 0.735 | **0.607** |
| Min | 0.385 | 0.289 | 0.458 | 0.385 | 0.220 | 0.189 | 0.144 | 0.106 | 0.578 | 0.427 | 0.523 | 0.422 | 0.535 | 0.427 |
| Max | 0.687 | 0.588 | 0.855 | 0.678 | 0.589 | 0.549 | 0.734 | 0.553 | 0.898 | 0.724 | 0.871 | 0.738 | 0.877 | 0.715 |

1. SR^1^stands for similarity reduced.

2. The Consensus-best3 method is based on NN-align, SMM-align and combinatorial peptide library. PROPRED was used for allelic variants when combinatorial peptide library was not available

**Table S2. The number of peptides selected with different similarity reduction approaches.**

The data is derived from ten repeated runs of each selection algorithms. The first number in each cell is the average number of selected peptides. The second number is the standard deviation. For cells with only one number, the standard deviation is zero.

| Allelic variant | ALL | Random  reduced | Forward  selection  reduced |
| --- | --- | --- | --- |
| HLA-DPA1*0103-DPB1*0201 | 1404 | 572.2±2.78 | 603 |
| HLA-DPA1*01-DPB1*0401 | 1337 | 511.8±2.74 | 540 |
| HLA-DPA1*0201-DPB1*0101 | 1399 | 571.7±3.50 | 604 |
| HLA-DPA1*0201-DPB1*0501 | 1410 | 558.1±5.47 | 586 |
| HLA-DPA1*0301-DPB1*0402 | 1407 | 572.5±3.37 | 602 |
| HLA-DQA1*0101-DQB1*0501 | 1739 | 563.1±4.61 | 584 |
| HLA-DQA1*0102-DQB1*0602 | 1629 | 561.5±4.30 | 593 |
| HLA-DQA1*0301-DQB1*0302 | 1719 | 570.7±3.43 | 596 |
| HLA-DQA1*0401-DQB1*0402 | 1701 | 555.5±4.99 | 585 |
| HLA-DQA1*0501-DQB1*0201 | 1658 | 558.8±4.13 | 589 |
| HLA-DQA1*0501-DQB1*0301 | 1689 | 572.4±4.01 | 602 |
| HLA-DRB1*0101 | 6427 | 3297.2±11.39 | 3504 |
| HLA-DRB1*0301 | 1715 | 1109.1±5.49 | 1136 |
| HLA-DRB1*0401 | 1769 | 1190.5±6.84 | 1221 |
| HLA-DRB1*0404 | 577 | 468.7±2.00 | 474 |
| HLA-DRB1*0405 | 1582 | 1015.8±3.29 | 1049 |
| HLA-DRB1*0701 | 1745 | 1142.1±5.69 | 1175 |
| HLA-DRB1*0802 | 1520 | 984.8±5.33 | 1017 |
| HLA-DRB1*0901 | 1520 | 1011.3±6.60 | 1042 |
| HLA-DRB1*1101 | 1794 | 1170.9±4.12 | 1204 |
| HLA-DRB1*1302 | 1580 | 1038.7±3.97 | 1070 |
| HLA-DRB1*1501 | 1769 | 1142.9±3.35 | 1171 |
| HLA-DRB3*0101 | 1501 | 958.5±5.84 | 987 |
| HLA-DRB4*0101 | 1521 | 974.1±3.31 | 1011 |
| HLA-DRB5*0101 | 1769 | 1168.4±5.52 | 1198 |
| H-2-IAb | 660 | 538.9±1.79 | 546 |

**Table S3. Performance of consensus approach with different combination of individual prediction algorithm.**

Best prediction performance for each allelic variant was highlighted in bold.

|  | all | no  ARB | no  comb^1^ | no  NN-align | no  SMM-align | no  PROPRED |
| --- | --- | --- | --- | --- | --- | --- |
| HLA-DPA1*0103-DPB1*0201 | 0.809 | 0.796 | 0.797 | 0.788 | **0.811** |  |
| HLA-DPA1*01-DPB1*0401 | **0.803** | 0.794 | **0.803** | 0.784 | 0.791 |  |
| HLA-DPA1*0201-DPB1*0101 | 0.818 | **0.819** | 0.811 | 0.794 | 0.805 |  |
| HLA-DPA1*0201-DPB1*0501 | 0.781 | **0.782** | 0.766 | 0.759 | 0.781 |  |
| HLA-DPA1*0301-DPB1*0402 | **0.841** | 0.830 | 0.834 | 0.835 | 0.832 |  |
| HLA-DQA1*0101-DQB1*0501 | 0.809 | **0.811** | 0.804 | 0.791 | 0.799 |  |
| HLA-DQA1*0102-DQB1*0602 | 0.778 | **0.779** | 0.761 | 0.760 | 0.774 |  |
| HLA-DQA1*0301-DQB1*0302 | 0.690 | **0.692** | 0.682 | 0.671 | 0.686 |  |
| HLA-DQA1*0401-DQB1*0402 | 0.749 | **0.762** | 0.750 | 0.731 | 0.713 |  |
| HLA-DQA1*0501-DQB1*0201 | 0.774 | **0.779** | 0.778 | 0.738 | 0.753 |  |
| HLA-DQA1*0501-DQB1*0301 | 0.814 | **0.816** | 0.810 | 0.800 | 0.807 |  |
| HLA-DRB1*0101 | 0.759 | 0.763 | 0.763 | 0.749 | 0.755 | 0.764 |
| HLA-DRB1*0301 | 0.823 | 0.823 |  | 0.788 | 0.797 | 0.831 |
| HLA-DRB1*0401 | 0.735 | **0.738** |  | 0.719 | 0.731 | 0.722 |
| HLA-DRB1*0404 | 0.800 | 0.807 |  | 0.776 | 0.787 | 0.794 |
| HLA-DRB1*0405 | **0.797** | 0.795 |  | 0.779 | 0.790 | 0.793 |
| HLA-DRB1*0701 | 0.806 | **0.810** | 0.807 | 0.804 | 0.806 | **0.810** |
| HLA-DRB1*0802 | 0.708 | 0.708 |  | 0.688 | 0.695 | 0.705 |
| HLA-DRB1*0901 | **0.716** | **0.716** | 0.710 | 0.692 | 0.702 |  |
| HLA-DRB1*1101 | 0.850 | 0.852 |  | 0.829 | 0.838 | 0.851 |
| HLA-DRB1*1302 | 0.742 | 0.751 |  | 0.719 | 0.699 | 0.751 |
| HLA-DRB1*1501 | 0.756 | **0.758** |  | 0.741 | 0.742 | 0.751 |
| HLA-DRB3*0101 | 0.787 | **0.799** | 0.789 | 0.751 | 0.763 |  |
| HLA-DRB4*0101 | **0.791** | 0.784 | 0.785 | 0.776 | 0.788 |  |
| HLA-DRB5*0101 | 0.786 | 0.791 |  | 0.761 | 0.771 | 0.785 |
| H-2-IAb | 0.846 | **0.847** |  | 0.826 | 0.834 |  |
| Average | 0.783 | 0.785 | 0.778 | 0.764 | 0.771 | 0.778 |
| t-test^2^ |  | 0.287 | 0.011 | <0.001 | <0.001 | 0.825 |

1. Comb stands for combinatorial peptide library.

2. Paired two tailed t-tests were performed to test no ARB, no comb, no SMM-align, no NN-align and no PROPRED versions of the consensus against the consensus based on all methods.

**Table S4. The measured IC50 values for the peptide mixtures for HLA-DPA1*0103-DPB1*0201**.

An “X” indicates random amino acids in that position for the peptide mixture.

| Peptide | IC50 (nM) |
| --- | --- |
| XXXXXXXXXXXXX | 69 |
| AAXXXXXXXXXAA | 56 |
| AAAXXXXXXXXAA | 83 |
| AACXXXXXXXXAA | 168 |
| AADXXXXXXXXAA | 193 |
| AAEXXXXXXXXAA | 221 |
| AAFXXXXXXXXAA | 56 |
| AAGXXXXXXXXAA | 155 |
| AAHXXXXXXXXAA | 24 |
| AAIXXXXXXXXAA | 59 |
| AAKXXXXXXXXAA | 37 |
| AALXXXXXXXXAA | 119 |
| AAMXXXXXXXXAA | 140 |
| AANXXXXXXXXAA | 204 |
| AAPXXXXXXXXAA | 199 |
| AAQXXXXXXXXAA | 106 |
| AARXXXXXXXXAA | 72 |
| AASXXXXXXXXAA | 106 |
| AATXXXXXXXXAA | 127 |
| AAVXXXXXXXXAA | 129 |
| AAWXXXXXXXXAA | 128 |
| AAYXXXXXXXXAA | 95 |
| AAXAXXXXXXXAA | 81 |
| AAXCXXXXXXXAA | 220 |
| AAXDXXXXXXXAA | 178 |
| AAXEXXXXXXXAA | 164 |
| AAXFXXXXXXXAA | 28 |
| AAXGXXXXXXXAA | 812 |
| AAXHXXXXXXXAA | 209 |
| AAXIXXXXXXXAA | 106 |
| AAXKXXXXXXXAA | 200 |
| AAXLXXXXXXXAA | 81 |
| AAXMXXXXXXXAA | 116 |
| AAXNXXXXXXXAA | 492 |
| AAXPXXXXXXXAA | 148 |
| AAXQXXXXXXXAA | 280 |
| AAXRXXXXXXXAA | 56 |
| AAXSXXXXXXXAA | 131 |
| AAXTXXXXXXXAA | 71 |
| AAXVXXXXXXXAA | 45 |
| AAXWXXXXXXXAA | 47 |
| AAXYXXXXXXXAA | 112 |
| AAXXAXXXXXXAA | 170 |
| AAXXCXXXXXXAA | 286 |
| AAXXDXXXXXXAA | 572 |
| AAXXEXXXXXXAA | 333 |
| AAXXFXXXXXXAA | 96 |
| AAXXGXXXXXXAA | 327 |
| AAXXHXXXXXXAA | 357 |
| AAXXIXXXXXXAA | 93 |
| AAXXKXXXXXXAA | 293 |
| AAXXLXXXXXXAA | 116 |
| AAXXMXXXXXXAA | 205 |
| AAXXNXXXXXXAA | 354 |
| AAXXPXXXXXXAA | 919 |
| AAXXQXXXXXXAA | 423 |
| AAXXRXXXXXXAA | 148 |
| AAXXSXXXXXXAA | 51 |
| AAXXTXXXXXXAA | 198 |
| AAXXVXXXXXXAA | 93 |
| AAXXWXXXXXXAA | 58 |
| AAXXYXXXXXXAA | 76 |
| AAXXXAXXXXXAA | 138 |
| AAXXXCXXXXXAA | 350 |
| AAXXXDXXXXXAA | 86 |
| AAXXXEXXXXXAA | 75 |
| AAXXXFXXXXXAA | 42 |
| AAXXXGXXXXXAA | 379 |
| AAXXXHXXXXXAA | 116 |
| AAXXXIXXXXXAA | 172 |
| AAXXXKXXXXXAA | 407 |
| AAXXXLXXXXXAA | 87 |
| AAXXXMXXXXXAA | 120 |
| AAXXXNXXXXXAA | 181 |
| AAXXXPXXXXXAA | 511 |
| AAXXXQXXXXXAA | 335 |
| AAXXXRXXXXXAA | 348 |
| AAXXXSXXXXXAA | 161 |
| AAXXXTXXXXXAA | 44 |
| AAXXXVXXXXXAA | 79 |
| AAXXXWXXXXXAA | 61 |
| AAXXXYXXXXXAA | 38 |
| AAXXXXAXXXXAA | 33 |
| AAXXXXCXXXXAA | 610 |
| AAXXXXDXXXXAA | 246 |
| AAXXXXEXXXXAA | 52 |
| AAXXXXFXXXXAA | 100 |
| AAXXXXGXXXXAA | 123 |
| AAXXXXHXXXXAA | 100 |
| AAXXXXIXXXXAA | 103 |
| AAXXXXKXXXXAA | 140 |
| AAXXXXLXXXXAA | 90 |
| AAXXXXMXXXXAA | 183 |
| AAXXXXNXXXXAA | 112 |
| AAXXXXPXXXXAA | 339 |
| AAXXXXQXXXXAA | 85 |
| AAXXXXRXXXXAA | 48 |
| AAXXXXSXXXXAA | 69 |
| AAXXXXTXXXXAA | 45 |
| AAXXXXVXXXXAA | 83 |
| AAXXXXWXXXXAA | 94 |
| AAXXXXYXXXXAA | 46 |
| AAXXXXXAXXXAA | 89 |
| AAXXXXXCXXXAA | 691 |
| AAXXXXXDXXXAA | 519 |
| AAXXXXXEXXXAA | 129 |
| AAXXXXXFXXXAA | 24 |
| AAXXXXXGXXXAA | 258 |
| AAXXXXXHXXXAA | 275 |
| AAXXXXXIXXXAA | 96 |
| AAXXXXXKXXXAA | 189 |
| AAXXXXXLXXXAA | 165 |
| AAXXXXXMXXXAA | 184 |
| AAXXXXXNXXXAA | 280 |
| AAXXXXXPXXXAA | 291 |
| AAXXXXXQXXXAA | 332 |
| AAXXXXXRXXXAA | 212 |
| AAXXXXXSXXXAA | 132 |
| AAXXXXXTXXXAA | 69 |
| AAXXXXXVXXXAA | 124 |
| AAXXXXXWXXXAA | 159 |
| AAXXXXXYXXXAA | 33 |
| AAXXXXXXAXXAA | 119 |
| AAXXXXXXCXXAA | 154 |
| AAXXXXXXDXXAA | 145 |
| AAXXXXXXEXXAA | 99 |
| AAXXXXXXFXXAA | 17 |
| AAXXXXXXGXXAA | 734 |
| AAXXXXXXHXXAA | 404 |
| AAXXXXXXIXXAA | 82 |
| AAXXXXXXKXXAA | 271 |
| AAXXXXXXLXXAA | 88 |
| AAXXXXXXMXXAA | 501 |
| AAXXXXXXNXXAA | 382 |
| AAXXXXXXPXXAA | 249 |
| AAXXXXXXQXXAA | 299 |
| AAXXXXXXRXXAA | 91 |
| AAXXXXXXSXXAA | 125 |
| AAXXXXXXTXXAA | 287 |
| AAXXXXXXVXXAA | 154 |
| AAXXXXXXWXXAA | 45 |
| AAXXXXXXYXXAA | 48 |
| AAXXXXXXXAXAA | 91 |
| AAXXXXXXXCXAA | 106 |
| AAXXXXXXXDXAA | 4057 |
| AAXXXXXXXEXAA | 168 |
| AAXXXXXXXFXAA | 33 |
| AAXXXXXXXGXAA | 316 |
| AAXXXXXXXHXAA | 216 |
| AAXXXXXXXIXAA | 40 |
| AAXXXXXXXKXAA | 74 |
| AAXXXXXXXLXAA | 20 |
| AAXXXXXXXMXAA | 240 |
| AAXXXXXXXNXAA | 534 |
| AAXXXXXXXPXAA | 460 |
| AAXXXXXXXQXAA | 110 |
| AAXXXXXXXRXAA | 121 |
| AAXXXXXXXSXAA | 231 |
| AAXXXXXXXTXAA | 130 |
| AAXXXXXXXVXAA | 155 |
| AAXXXXXXXWXAA | 85 |
| AAXXXXXXXYXAA | 91 |
| AAXXXXXXXXAAA | 129 |
| AAXXXXXXXXCAA | 223 |
| AAXXXXXXXXDAA | 207 |
| AAXXXXXXXXEAA | 136 |
| AAXXXXXXXXFAA | 83 |
| AAXXXXXXXXGAA | 1427 |
| AAXXXXXXXXHAA | 520 |
| AAXXXXXXXXIAA | 16 |
| AAXXXXXXXXKAA | 96 |
| AAXXXXXXXXLAA | 54 |
| AAXXXXXXXXMAA | 325 |
| AAXXXXXXXXNAA | 239 |
| AAXXXXXXXXPAA | 412 |
| AAXXXXXXXXQAA | 137 |
| AAXXXXXXXXRAA | 172 |
| AAXXXXXXXXSAA | 129 |
| AAXXXXXXXXTAA | 77 |
| AAXXXXXXXXVAA | 70 |
| AAXXXXXXXXWAA | 259 |
| AAXXXXXXXXYAA | 157 |

**Table S5. The scoring matrix of HLA-DPA1*0103-DPB1*0201 derived from the IC50 values of peptide mixtures listed in Supplementary Table 4.**

| Amino Acid | P1 | P2 | P3 | P4 | P5 | P6 | P7 | P8 | P9 |
| --- | --- | --- | --- | --- | --- | --- | --- | --- | --- |
| A | 0.10 | 0.20 | 0.06 | 0.00 | 0.49 | 0.26 | 0.10 | 0.21 | 0.09 |
| C | -0.20 | -0.24 | -0.17 | -0.41 | -0.78 | -0.63 | -0.01 | 0.14 | -0.15 |
| D | -0.26 | -0.15 | -0.47 | 0.20 | -0.38 | -0.51 | 0.02 | -1.44 | -0.12 |
| E | -0.32 | -0.11 | -0.24 | 0.27 | 0.29 | 0.10 | 0.19 | -0.06 | 0.06 |
| F | 0.27 | 0.65 | 0.31 | 0.51 | 0.01 | 0.82 | 0.95 | 0.65 | 0.28 |
| G | -0.17 | -0.81 | -0.23 | -0.44 | -0.08 | -0.21 | -0.69 | -0.33 | -0.96 |
| H | 0.65 | -0.22 | -0.26 | 0.07 | 0.01 | -0.23 | -0.43 | -0.16 | -0.52 |
| I | 0.25 | 0.08 | 0.32 | -0.10 | -0.01 | 0.23 | 0.26 | 0.56 | 0.98 |
| K | 0.46 | -0.20 | -0.18 | -0.47 | -0.14 | -0.07 | -0.25 | 0.30 | 0.22 |
| L | -0.05 | 0.19 | 0.22 | 0.20 | 0.05 | -0.01 | 0.23 | 0.87 | 0.47 |
| M | -0.12 | 0.04 | -0.02 | 0.06 | -0.26 | -0.06 | -0.52 | -0.21 | -0.31 |
| N | -0.29 | -0.59 | -0.26 | -0.12 | -0.04 | -0.24 | -0.40 | -0.56 | -0.18 |
| P | -0.28 | -0.07 | -0.68 | -0.57 | -0.52 | -0.26 | -0.22 | -0.49 | -0.42 |
| Q | 0.00 | -0.34 | -0.34 | -0.39 | 0.08 | -0.31 | -0.29 | 0.13 | 0.06 |
| R | 0.16 | 0.36 | 0.12 | -0.40 | 0.32 | -0.12 | 0.22 | 0.09 | -0.04 |
| S | 0.00 | -0.01 | 0.58 | -0.07 | 0.17 | 0.09 | 0.08 | -0.19 | 0.09 |
| T | -0.08 | 0.25 | -0.01 | 0.50 | 0.35 | 0.37 | -0.28 | 0.06 | 0.31 |
| V | -0.09 | 0.46 | 0.32 | 0.24 | 0.09 | 0.11 | -0.01 | -0.02 | 0.35 |
| W | -0.08 | 0.43 | 0.52 | 0.35 | 0.03 | 0.01 | 0.53 | 0.24 | -0.22 |
| Y | 0.04 | 0.06 | 0.41 | 0.56 | 0.34 | 0.68 | 0.50 | 0.21 | 0.00 |
